# (4-Eth­oxy­benzo­yl)[8-(4-eth­oxy­benzo­yl)-2,7-di­meth­oxy­naphthalen-1-yl]methanone

**DOI:** 10.1107/S1600536813008581

**Published:** 2013-04-05

**Authors:** Kosuke Sasagawa, Rei Sakamoto, Daichi Hijikata, Noriyuki Yonezawa, Akiko Okamoto

**Affiliations:** a Department of Organic and Polymer Materials Chemistry, Tokyo University of Agriculture & Technology, Koganei, Tokyo 184-8588, Japan

## Abstract

The title mol­ecule, C_30_H_28_O_6_, possesses crystallographically imposed twofold symmetry, with two central C atoms in the naphthalene unit lying on the rotation axis along [001]. The 4-eth­oxy­benzoyl groups at the *peri* positions of the naphthalene ring system are disordered over two sets of sites with occupancies of 0.769 (4) and 0.231 (4). They are directed in opposite directions from the naphthalene plane (*anti* orientation). For the major component, the dihedral angle between the aroyl benzene ring and the naphthalene ring system is 75.62 (13)° [minor component 75.5 (4)°], and that between the aroyl benzene rings is 32.58 (15)°. In the crystal, mol­ecules are linked *via* C—H⋯O and C—H⋯π inter­actions, forming a three-dimensional network.

## Related literature

For formation reactions of aroylated naphthalene compounds *via* electrophilic aromatic substitution of naphthalene derivatives, see: Okamoto & Yonezawa (2009 ▶); Okamoto *et al.* (2011 ▶). For the structures of closely related compounds, see: Hijikata *et al.* (2010 ▶); Sasagawa *et al.* (2011 ▶, 2012 ▶); Sasagawa, Sakamoto *et al.* (2013 ▶); Sasagawa, Takeuchi *et al.* (2013 ▶).
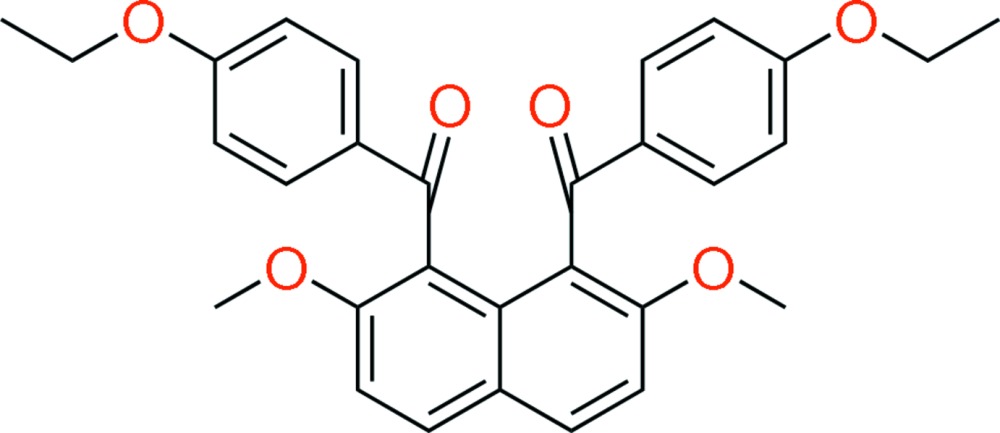


## Experimental

### 

#### Crystal data

C_30_H_28_O_6_
*M* *_r_* = 484.52
Orthorhombic, 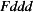

*a* = 19.6446 (4) Å
*b* = 21.5251 (4) Å
*c* = 22.9585 (4) Å
*V* = 9708.0 (3) Å^3^
*Z* = 16
Cu *K*α radiation
μ = 0.75 mm^−1^
*T* = 193 K
0.60 × 0.50 × 0.50 mm

#### Data collection

Rigaku R-AXIS RAPID diffractometer
Absorption correction: numerical (*NUMABS*; Higashi, 1999 ▶) *T*_min_ = 0.662, *T*_max_ = 0.706
40441 measured reflections
2230 independent reflections
2113 reflections with *I* > 2σ(*I*)
*R*_int_ = 0.025

#### Refinement

*R*[*F* ^2^ > 2σ(*F* ^2^)] = 0.041
*wR*(*F* ^2^) = 0.099
*S* = 1.15
2230 reflections
238 parameters
20 restraints
H-atom parameters constrained
Δρ_max_ = 0.20 e Å^−3^
Δρ_min_ = −0.31 e Å^−3^

### 

Data collection: *PROCESS-AUTO* (Rigaku, 1998 ▶); cell refinement: *PROCESS-AUTO*; data reduction: *PROCESS-AUTO*; program(s) used to solve structure: *SHELXS97* (Sheldrick, 2008 ▶); program(s) used to refine structure: *SHELXL97* (Sheldrick, 2008 ▶); molecular graphics: *ORTEPIII* (Burnett & Johnson, 1996 ▶); software used to prepare material for publication: *SHELXL97*.

## Supplementary Material

Crystal structure: contains datablock(s) I, global. DOI: 10.1107/S1600536813008581/gk2562sup1.cif

Structure factors: contains datablock(s) I. DOI: 10.1107/S1600536813008581/gk2562Isup2.hkl

Supplementary material file. DOI: 10.1107/S1600536813008581/gk2562Isup3.cml

Additional supplementary materials:  crystallographic information; 3D view; checkCIF report

## Figures and Tables

**Table 1:** Hydrogen-bond geometry (Å, °) *Cg*1 and *Cg*2 are the centroids of the C8–C13 and C1-C6 rings, respectively

*D*—H⋯*A*	*D*—H	H⋯*A*	*D*⋯*A*	*D*—H⋯*A*
C16—H16*C*⋯O1^i^	0.98	2.51	3.4919 (16)	175
C14—H14*C*⋯*Cg*1^i^	0.98	2.83	3.716 (2)	151
C15—H15*B*⋯*Cg*2^i^	0.99	2.80	3.6831 (19)	149

Symmetry code: (i) 
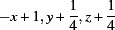
.

**Fig. 1.**
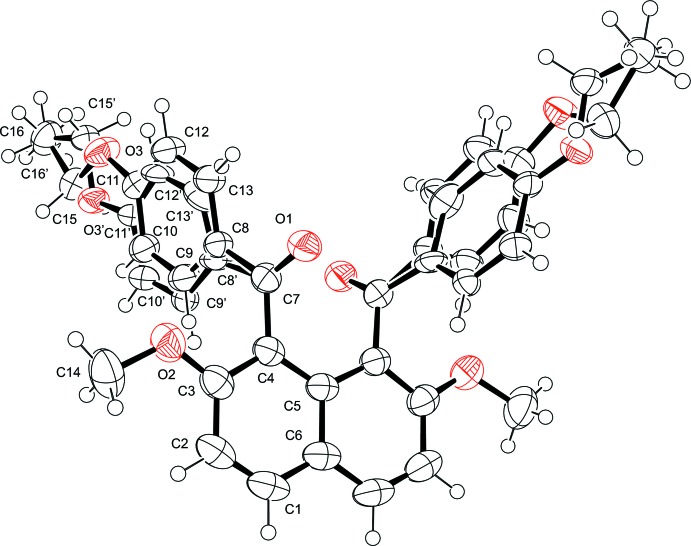
Molecular structure with displacement ellipsoids drawn at the 50% probability level. Symmetry code for generation of unlabeled atoms: -x + 5/4, -y + 1/4, z.

**Fig. 2.**
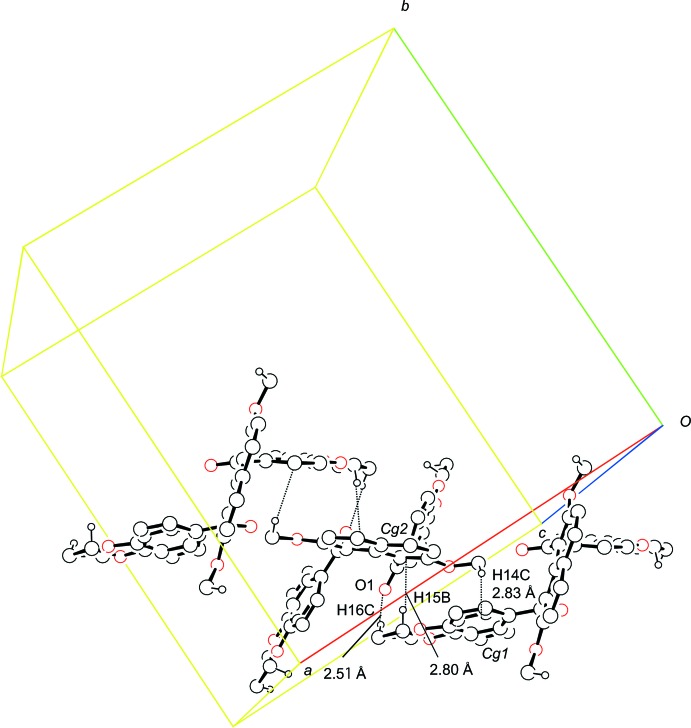
Intermolecular C—H···O interaction between the methyl group in the ethoxy group and the ketonic carbonyl group, C—H···π interaction between the methoxy group and the benzene ring, and that between the methylene moiety of the ethoxy group and the naphthalene group [symmetry code: -x + 1, y + 1/4, z + 1/4 along the a axis (dashed lines).

## supplementary crystallographic information

### Comment

In the course of our study on selective electrophilic aromatic aroylation of the naphthalene ring core, 1,8-diaroylnaphthalene compounds have proved to be formed regioselectively by the aid of a suitable acidic mediator (Okamoto & Yonezawa, 2009, Okamoto *et al.*, 2011). Recently, we have reported the X-ray crystal structures of 1,8-diaroylated 2,7-dimethoxynaphthalene derivatives such as 1,8-bis(4-butoxylbenzoyl)-2,7-dimethoxynaphthalene [{8-[4-(butoxy)benzoyl]-2,7-dimethoxynaphthalen-1-yl}[4-(butoxy)phenyl]methanone] (Sasagawa *et al.*, 2011), 1,8-bis(4-methoxybenzoyl)-2,7-dimethoxynaphthalene [{2,7-dimethoxy-8-(4-methoxybenzoyl)-naphthalen-1-yl}(4-methoxyphenyl)-methanone chloroform monosolvate] (Sasagawa, Sakamoto *et al.*, 2013) and 1,8-bis(4-isobutylbenzoyl)-2,7-dimethoxynaphthalene [{2,7-dimethoxy-8-[4-(2-methylpropyl)benzoyl]-naphthalen-1-yl}{4-(2-methylpropyl)phenyl-methanone}] (Sasagawa *et al.*, 2012).

The aroyl groups in these compounds are almost perpendicularly attached to the naphthalene rings and oriented in opposite directions (*anti*-orientation). According to the authors' knowledge, most 1,8-diaroylnaphthalene derivatives have *anti*-oriented structures. Recently, we have also clarified another structure of the 1,8-diaroylnaphthalene derivatives, where the two aroyl groups are situated in same direction (*syn*-orientation), 2,7-dimethoxy-1,8-bis(4-phenoxybenzoyl)naphthalene (Hijikata *et al.*, 2010) and 1,8-bis(4-isopropoxybenzoyl)-2,7-dimethoxynaphthalene [{2,7-dimethoxy-8-[4-(propan-2-yloxy)-benzoyl]naphthalen-1-yl}[4-(propan-2-yloxy)phenyl]methanone] (Sasagawa, Takeuchi *et al.*, 2013). As a part of our ongoing studies on the molecular structures of these kinds of homologous molecules, the X-ray crystal structure of the title compound, 1,8-diaroylated naphthalene bearing ethoxy groups on the aroyl moieties, is discussed herein.

The molecular structure of the title compound is displayed in Fig 1. The title molecule lies on a crystallographic twofold axis so that the asymmetric unit contains one-half of the molecule. Thus, two 4-ethoxybenzoyl groups are situated in *anti*-orientation and are twisted away from the attached naphthalene ring. The dihedral angle between the best planes of the 4-ethoxyphenyl groups and the naphthalene ring system is 75.62 (13)° [or 75.5 (4)° for minor position].

The torsion angles along the bond between the naphthalene ring system and the bridging carbonyl moiety, C2—C1—C7—O1, is -109.85 (12)° , whereas that between the phenyl group and the bridging carbonyl moiety (O1—C7—C8—C9) is -169.0 (4)°.

In the crystal, C—H···O and two kinds of C—H···π interactions link the molecules and form a three-dimensional network (Table 1, Fig 2; symmetry code: -*x* + 1, *y* + 1/4, *z* + 1/4): a C—H···O interaction between a hydrogen atom of the methyl moiety in the ethoxy group and the oxygen atom of the carbonyl moiety (C16—H16···O1 = 2.51 Å), a C—H···π interaction between a hydrogen atom of the methoxy group and the π-system of benzene ring (C14—H14C···*Cg1* = 2.83 Å; *Cg1* is the centroid of C8-C13 ring), and a C—H···π interactions between a hydrogen atom of the methylene moiety in the ethoxy group and the *π*-system of naphthalene ring (C15—H15B···*Cg2* = 2.80 Å; *Cg2* is the centroid of C1-C6 ring).

### Experimental

The title compound was prepared by S_N_2 reaction of 1,8-bis(4-hydroxybenzoyl)-2,7-dimethoxynaphthalene (2.0 mmol, 857 mg), which was obtained *via* S_N_Ar reaction of 1,8-bis(4-fluorobenzoyl)-2,7-dimethoxynaphthalene with sodium hydroxide, with ethyl iodide (6.0 mmol, 936 mg) and potassium carbonate (5.6 mmol, 774 mg) in *N*,*N*-dimethylformamide (DMF; 5.0 ml). After the reaction mixture was stirred at 328 K for 6 h, it was poured into water (30 ml) and the mixture was extracted with CHCl_3_ (10 ml × 3). The combined extracts were washed with brine. The organic layers thus obtained were dried over anhydrous MgSO_4_. The solvent was removed under reduced pressure to give cake (93% yield). The crude product was purified by recrystallization from ethyl acetate (isolated yield 56%). Furthermore, the isolated product was crystallized from ethyl acetate to give single crystal. (m.p. = 473.6—477.6 K). Spectroscopic data for the title compound is available in the archived CIF.

### Refinement

All H atoms were found in a difference map and were subsequently refined as riding atoms, with C—H = 0.95 (aromatic), 0.98 (methyl) and 0.99 (methylene) Å with *U*_ĩso_(H) = 1.2*U*_eq_(C) or 1.5 *U*_eq_(C). The 4-ethoxybenzene group is disordered over two positions with atoms C7/C7' and C16/C16' completely overlapping. The coordinates and anisotropic displacement parameters of these atomic pairs were constrained with EXYZ and EADP instructions of SHELXL-97 (Sheldrick, 2008). Moreover restraints were imposed on the geometry of the minor orientation with the instruction SAME. The occupancies of the two postions refined at 0.769 (4) and 0.231 (4).

### Crystal data

**Table:**

C_30_H_28_O_6_	*F*(000) = 4096
*M**_r_* = 484.52	*D*_x_ = 1.326 Mg m^−^^3^
Orthorhombic, *F**d**d**d*	Cu *K*α radiation, λ = 1.54187 Å
Hall symbol: -F 2uv 2vw	Cell parameters from 37043 reflections
*a* = 19.6446 (4) Å	θ = 3.6–68.2°
*b* = 21.5251 (4) Å	µ = 0.75 mm^−^^1^
*c* = 22.9585 (4) Å	*T* = 193 K
*V* = 9708.0 (3) Å^3^	Block, colorless
*Z* = 16	0.60 × 0.50 × 0.50 mm

### Data collection

**Table:**

Rigaku R-AXIS RAPID diffractometer	2230 independent reflections
Radiation source: fine-focus sealed tube	2113 reflections with *I* > 2σ(*I*)
Graphite monochromator	*R*_int_ = 0.025
Detector resolution: 10.000 pixels mm^-1^	θ_max_ = 68.2°, θ_min_ = 3.6°
ω scans	*h* = −23→23
Absorption correction: numerical (*NUMABS*; Higashi, 1999)	*k* = −25→25
*T*_min_ = 0.662, *T*_max_ = 0.706	*l* = −27→27
40441 measured reflections	

### Refinement

**Table:**

Refinement on *F*^2^	Primary atom site location: structure-invariant direct methods
Least-squares matrix: full	Secondary atom site location: difference Fourier map
*R*[*F*^2^ > 2σ(*F*^2^)] = 0.041	Hydrogen site location: inferred from neighbouring sites
*wR*(*F*^2^) = 0.099	H-atom parameters constrained
*S* = 1.15	*w* = 1/[σ^2^(*F*_o_^2^) + (0.0506*P*)^2^ + 5.2333*P*] where *P* = (*F*_o_^2^ + 2*F*_c_^2^)/3
2230 reflections	(Δ/σ)_max_ = 0.001
238 parameters	Δρ_max_ = 0.20 e Å^−^^3^
20 restraints	Δρ_min_ = −0.31 e Å^−^^3^

### Special details

**Table:**

Experimental. Spectroscopic Data: ^1^H NMR δ (300 MHz, CDCl_3_): 1.42 (6H, t, *J* = 7.2 Hz), 3.71 (6H, s), 4.05 (4H, q, *J* = 7.2 Hz), 6.78 (4H, br), 7.20 (2H, d, *J* = 9.3 Hz), 7.64 (4H, br), 7.92 (2H, d, *J* = 9.3 Hz) p.p.m ^13^C NMR δ (75 MHz, CDCl_3_): 14.7, 56.4, 63.4, 111.2, 113.5, 121.8, 125.6, 129.6, 131.3, 131.6, 131.9, 155.9, 162.4, 194.9 p.p.m IR (KBr): 2980 (CH_3_), 2938 (CH_2_), 1658 (C=O), 1601, 1511, 1474 (Ar) cm^-1^ HRMS (m/z): [M+H]^+^ calcd. for C_30_H_29_O_6_, 485.1964 , found, 485.1995m.p. = 473.6—477.6 K
Geometry. All s.u.'s (except the s.u. in the dihedral angle between two l.s. planes) are estimated using the full covariance matrix. The cell s.u.'s are taken into account individually in the estimation of s.u.'s in distances, angles and torsion angles; correlations between s.u.'s in cell parameters are only used when they are defined by crystal symmetry. An approximate (isotropic) treatment of cell s.u.'s is used for estimating s.u.'s involving l.s. planes.
Refinement. Refinement of *F*^2^ against ALL reflections. The weighted *R*-factor *wR* and goodness of fit *S* are based on *F*^2^, conventional *R*-factors *R* are based on *F*, with *F* set to zero for negative *F*^2^. The threshold expression of *F*^2^ > 2σ(*F*^2^) is used only for calculating *R*-factors(gt) *etc*. and is not relevant to the choice of reflections for refinement. *R*-factors based on *F*^2^ are statistically about twice as large as those based on *F*, and *R*- factors based on ALL data will be even larger.

### Fractional atomic coordinates and isotropic or equivalent isotropic displacement parameters (Å^2^)

**Table:**

	*x*	*y*	*z*	*U*_iso_*/*U*_eq_	Occ. (<1)
O1	0.62404 (4)	0.04943 (4)	0.43348 (3)	0.0512 (2)	
O2	0.49033 (5)	0.01158 (5)	0.34362 (4)	0.0625 (3)	
C1	0.57676 (5)	0.08569 (5)	0.34527 (5)	0.0422 (3)	
C2	0.53143 (6)	0.05038 (6)	0.31286 (5)	0.0496 (3)	
C3	0.53080 (7)	0.05265 (6)	0.25136 (5)	0.0569 (3)	
H3	0.4983	0.0294	0.2297	0.068*	
C4	0.57734 (7)	0.08853 (6)	0.22388 (5)	0.0568 (4)	
H4	0.5778	0.0891	0.1825	0.068*	
C5	0.6250	0.1250	0.25413 (7)	0.0485 (4)	
C6	0.6250	0.1250	0.31675 (6)	0.0415 (3)	
C7	0.57588 (5)	0.07524 (5)	0.41049 (5)	0.0420 (3)	0.769 (4)
C8	0.5170 (3)	0.0946 (3)	0.4472 (2)	0.0379 (8)	0.769 (4)
C9	0.4673 (2)	0.1329 (2)	0.42483 (14)	0.0446 (7)	0.769 (4)
H9	0.4697	0.1451	0.3851	0.054*	0.769 (4)
C10	0.41390 (10)	0.15410 (10)	0.45895 (16)	0.0444 (6)	0.769 (4)
H10	0.3803	0.1809	0.4431	0.053*	0.769 (4)
C11	0.41044 (13)	0.13542 (12)	0.51683 (15)	0.0430 (5)	0.769 (4)
C12	0.45874 (12)	0.09486 (11)	0.53927 (10)	0.0485 (6)	0.769 (4)
H12	0.4552	0.0807	0.5784	0.058*	0.769 (4)
C13	0.51175 (15)	0.07517 (14)	0.50485 (12)	0.0431 (6)	0.769 (4)
H13	0.5452	0.0480	0.5206	0.052*	0.769 (4)
O3	0.36167 (7)	0.15461 (6)	0.55517 (6)	0.0562 (5)	0.769 (4)
C15	0.31301 (10)	0.19884 (8)	0.53540 (10)	0.0497 (5)	0.769 (4)
H15A	0.2836	0.1803	0.5050	0.060*	0.769 (4)
H15B	0.3362	0.2356	0.5188	0.060*	0.769 (4)
C16	0.27090 (7)	0.21734 (7)	0.58794 (6)	0.0606 (4)	0.769 (4)
H16A	0.2509	0.1801	0.6056	0.091*	0.769 (4)
H16B	0.2345	0.2456	0.5757	0.091*	0.769 (4)
H16C	0.3001	0.2382	0.6165	0.091*	0.769 (4)
C14	0.43576 (8)	−0.01802 (9)	0.31315 (8)	0.0809 (5)	
H14A	0.4035	0.0135	0.2994	0.121*	
H14B	0.4123	−0.0469	0.3394	0.121*	
H14C	0.4540	−0.0410	0.2797	0.121*	
C7'	0.57588 (5)	0.07524 (5)	0.41049 (5)	0.0420 (3)	0.231 (4)
C8'	0.5121 (10)	0.1018 (12)	0.4338 (7)	0.041 (3)	0.231 (4)
C9'	0.4664 (6)	0.1408 (6)	0.4061 (4)	0.036 (2)	0.231 (4)
H9'	0.4743	0.1505	0.3663	0.044*	0.231 (4)
C10'	0.4103 (3)	0.1663 (3)	0.4325 (4)	0.0432 (18)	0.231 (4)
H10'	0.3802	0.1926	0.4115	0.052*	0.231 (4)
C11'	0.3988 (3)	0.1525 (3)	0.4911 (5)	0.0376 (15)	0.231 (4)
C12'	0.4409 (4)	0.1139 (4)	0.5219 (3)	0.0413 (17)	0.231 (4)
H12'	0.4319	0.1036	0.5614	0.050*	0.231 (4)
C13'	0.4977 (5)	0.0900 (5)	0.4926 (5)	0.052 (3)	0.231 (4)
H13'	0.5283	0.0644	0.5138	0.062*	0.231 (4)
O3'	0.3435 (2)	0.1813 (2)	0.51409 (16)	0.0478 (14)	0.231 (4)
C15'	0.3322 (3)	0.1786 (3)	0.5762 (2)	0.0493 (17)	0.231 (4)
H15C	0.3721	0.1951	0.5974	0.059*	0.231 (4)
H15D	0.3243	0.1352	0.5887	0.059*	0.231 (4)
C16'	0.27090 (7)	0.21734 (7)	0.58794 (6)	0.0606 (4)	0.231 (4)
H16D	0.2791	0.2599	0.5746	0.091*	0.231 (4)
H16E	0.2615	0.2176	0.6299	0.091*	0.231 (4)
H16F	0.2317	0.2000	0.5671	0.091*	0.231 (4)

### Atomic displacement parameters (Å^2^)

**Table:**

	*U*^11^	*U*^22^	*U*^33^	*U*^12^	*U*^13^	*U*^23^
O1	0.0481 (5)	0.0616 (5)	0.0440 (4)	0.0054 (4)	−0.0029 (3)	0.0054 (4)
O2	0.0588 (5)	0.0675 (6)	0.0612 (6)	−0.0130 (4)	−0.0051 (4)	−0.0125 (4)
C1	0.0418 (6)	0.0462 (6)	0.0386 (6)	0.0093 (5)	−0.0023 (4)	−0.0038 (4)
C2	0.0472 (6)	0.0521 (7)	0.0493 (6)	0.0085 (5)	−0.0060 (5)	−0.0076 (5)
C3	0.0621 (7)	0.0598 (8)	0.0489 (7)	0.0135 (6)	−0.0169 (6)	−0.0130 (6)
C4	0.0716 (8)	0.0628 (8)	0.0360 (6)	0.0231 (7)	−0.0098 (6)	−0.0067 (5)
C5	0.0581 (9)	0.0513 (9)	0.0361 (8)	0.0211 (8)	0.000	0.000
C6	0.0434 (8)	0.0459 (8)	0.0351 (7)	0.0150 (6)	0.000	0.000
C7	0.0422 (6)	0.0431 (6)	0.0407 (6)	−0.0017 (4)	−0.0011 (4)	−0.0020 (4)
C8	0.0411 (12)	0.0420 (16)	0.031 (2)	−0.0027 (8)	0.0015 (16)	−0.0001 (18)
C9	0.0487 (13)	0.0489 (14)	0.0362 (16)	0.0005 (10)	0.0024 (13)	0.0050 (13)
C10	0.0433 (10)	0.0475 (10)	0.0423 (17)	0.0031 (7)	0.0035 (12)	0.0059 (12)
C11	0.0444 (13)	0.0454 (13)	0.0393 (14)	−0.0013 (9)	0.0061 (13)	0.0015 (11)
C12	0.0561 (12)	0.0547 (12)	0.0347 (11)	0.0017 (9)	0.0047 (9)	0.0065 (8)
C13	0.0458 (12)	0.0476 (14)	0.0361 (12)	0.0041 (9)	−0.0003 (9)	0.0026 (9)
O3	0.0581 (8)	0.0647 (8)	0.0458 (8)	0.0093 (6)	0.0157 (6)	0.0059 (6)
C15	0.0448 (10)	0.0496 (9)	0.0546 (11)	−0.0029 (7)	0.0069 (9)	0.0007 (8)
C16	0.0530 (7)	0.0703 (8)	0.0584 (7)	−0.0021 (6)	0.0148 (6)	−0.0092 (6)
C14	0.0606 (9)	0.0851 (11)	0.0969 (12)	−0.0146 (8)	−0.0120 (8)	−0.0230 (9)
C7'	0.0422 (6)	0.0431 (6)	0.0407 (6)	−0.0017 (4)	−0.0011 (4)	−0.0020 (4)
C8'	0.056 (6)	0.049 (7)	0.018 (5)	−0.011 (4)	−0.005 (4)	0.005 (4)
C9'	0.033 (3)	0.043 (4)	0.033 (5)	0.005 (2)	0.001 (3)	0.000 (4)
C10'	0.046 (3)	0.052 (4)	0.031 (4)	0.000 (2)	−0.003 (3)	0.007 (3)
C11'	0.039 (4)	0.047 (3)	0.027 (4)	−0.003 (3)	−0.004 (3)	0.008 (3)
C12'	0.047 (4)	0.053 (5)	0.024 (3)	0.010 (3)	−0.004 (3)	0.017 (3)
C13'	0.054 (5)	0.046 (5)	0.055 (6)	0.007 (3)	−0.022 (4)	0.015 (3)
O3'	0.042 (2)	0.070 (3)	0.032 (2)	0.009 (2)	0.0069 (17)	−0.0009 (18)
C15'	0.049 (3)	0.068 (4)	0.031 (3)	−0.015 (3)	0.002 (2)	0.001 (3)
C16'	0.0530 (7)	0.0703 (8)	0.0584 (7)	−0.0021 (6)	0.0148 (6)	−0.0092 (6)

### Geometric parameters (Å, º)

**Table:**

O1—C7	1.2175 (13)	C13—H13	0.9500
O2—C2	1.3595 (16)	O3—C15	1.423 (3)
O2—C14	1.4300 (16)	C15—C16	1.516 (3)
C1—C2	1.3871 (16)	C15—H15A	0.9900
C1—C6	1.4293 (13)	C15—H15B	0.9900
C1—C7	1.5141 (15)	C16—H16A	0.9800
C2—C3	1.4130 (17)	C16—H16B	0.9800
C3—C4	1.353 (2)	C16—H16C	0.9800
C3—H3	0.9500	C14—H14A	0.9800
C4—C5	1.4054 (15)	C14—H14B	0.9800
C4—H4	0.9500	C14—H14C	0.9800
C5—C4^i^	1.4054 (15)	C8'—C9'	1.384 (12)
C5—C6	1.438 (2)	C8'—C13'	1.402 (12)
C6—C1^i^	1.4293 (13)	C9'—C10'	1.372 (12)
C7—C8	1.492 (3)	C9'—H9'	0.9500
C8—C9	1.377 (4)	C10'—C11'	1.395 (9)
C8—C13	1.391 (4)	C10'—H10'	0.9500
C9—C10	1.387 (4)	C11'—O3'	1.357 (8)
C9—H9	0.9500	C11'—C12'	1.371 (8)
C10—C11	1.390 (3)	C12'—C13'	1.401 (12)
C10—H10	0.9500	C12'—H12'	0.9500
C11—O3	1.365 (3)	C13'—H13'	0.9500
C11—C12	1.389 (3)	O3'—C15'	1.443 (7)
C12—C13	1.374 (4)	C15'—H15C	0.9900
C12—H12	0.9500	C15'—H15D	0.9900
			
C2—O2—C14	117.70 (12)	C12—C13—C8	120.7 (3)
C2—C1—C6	120.28 (11)	C12—C13—H13	119.7
C2—C1—C7	116.22 (10)	C8—C13—H13	119.7
C6—C1—C7	123.27 (10)	C11—O3—C15	117.9 (3)
O2—C2—C1	116.05 (11)	O3—C15—C16	106.77 (16)
O2—C2—C3	122.35 (11)	O3—C15—H15A	110.4
C1—C2—C3	121.52 (12)	C16—C15—H15A	110.4
C4—C3—C2	118.67 (12)	O3—C15—H15B	110.4
C4—C3—H3	120.7	C16—C15—H15B	110.4
C2—C3—H3	120.7	H15A—C15—H15B	108.6
C3—C4—C5	122.59 (11)	C15—C16—H16A	109.5
C3—C4—H4	118.7	C15—C16—H16B	109.5
C5—C4—H4	118.7	H16A—C16—H16B	109.5
C4—C5—C4^i^	120.77 (15)	C15—C16—H16C	109.5
C4—C5—C6	119.61 (8)	H16A—C16—H16C	109.5
C4^i^—C5—C6	119.61 (8)	H16B—C16—H16C	109.5
C1—C6—C1^i^	125.46 (13)	C9'—C8'—C13'	114.9 (10)
C1—C6—C5	117.27 (7)	C10'—C9'—C8'	124.2 (9)
C1^i^—C6—C5	117.27 (7)	C10'—C9'—H9'	117.9
O1—C7—C8	119.0 (2)	C8'—C9'—H9'	117.9
O1—C7—C1	119.18 (10)	C9'—C10'—C11'	118.1 (6)
C8—C7—C1	121.8 (2)	C9'—C10'—H10'	121.0
C9—C8—C13	118.9 (3)	C11'—C10'—H10'	121.0
C9—C8—C7	120.4 (3)	O3'—C11'—C12'	124.0 (9)
C13—C8—C7	120.7 (3)	O3'—C11'—C10'	114.1 (7)
C8—C9—C10	121.5 (3)	C12'—C11'—C10'	121.9 (6)
C8—C9—H9	119.3	C11'—C12'—C13'	117.1 (6)
C10—C9—H9	119.3	C11'—C12'—H12'	121.5
C9—C10—C11	118.8 (2)	C13'—C12'—H12'	121.5
C9—C10—H10	120.6	C12'—C13'—C8'	123.9 (8)
C11—C10—H10	120.6	C12'—C13'—H13'	118.1
O3—C11—C12	115.5 (3)	C8'—C13'—H13'	118.1
O3—C11—C10	124.3 (3)	C11'—O3'—C15'	119.3 (7)
C12—C11—C10	120.21 (18)	O3'—C15'—H15C	110.5
C13—C12—C11	119.9 (2)	O3'—C15'—H15D	110.5
C13—C12—H12	120.1	H15C—C15'—H15D	108.7
C11—C12—H12	120.1		
			
C14—O2—C2—C1	170.15 (11)	O1—C7—C8—C13	−10.0 (8)
C14—O2—C2—C3	−12.94 (18)	C1—C7—C8—C13	168.8 (5)
C6—C1—C2—O2	176.11 (8)	C13—C8—C9—C10	2.3 (10)
C7—C1—C2—O2	1.54 (14)	C7—C8—C9—C10	−176.7 (4)
C6—C1—C2—C3	−0.83 (16)	C8—C9—C10—C11	−0.7 (7)
C7—C1—C2—C3	−175.40 (11)	C9—C10—C11—O3	177.9 (3)
O2—C2—C3—C4	−174.32 (11)	C9—C10—C11—C12	−1.8 (4)
C1—C2—C3—C4	2.42 (18)	O3—C11—C12—C13	−177.0 (2)
C2—C3—C4—C5	−1.88 (17)	C10—C11—C12—C13	2.7 (3)
C3—C4—C5—C4^i^	179.78 (13)	C11—C12—C13—C8	−1.2 (6)
C3—C4—C5—C6	−0.22 (13)	C9—C8—C13—C12	−1.3 (10)
C2—C1—C6—C1^i^	178.75 (11)	C7—C8—C13—C12	177.6 (4)
C7—C1—C6—C1^i^	−7.09 (7)	C12—C11—O3—C15	176.67 (16)
C2—C1—C6—C5	−1.25 (11)	C10—C11—O3—C15	−3.0 (3)
C7—C1—C6—C5	172.91 (7)	C11—O3—C15—C16	−173.45 (13)
C4—C5—C6—C1	1.79 (7)	C13'—C8'—C9'—C10'	−1 (4)
C4^i^—C5—C6—C1	−178.21 (7)	C8'—C9'—C10'—C11'	0 (2)
C4—C5—C6—C1^i^	−178.21 (7)	C9'—C10'—C11'—O3'	178.0 (8)
C4^i^—C5—C6—C1^i^	1.79 (7)	C9'—C10'—C11'—C12'	−1.2 (12)
C2—C1—C7—O1	109.85 (12)	O3'—C11'—C12'—C13'	−177.1 (7)
C6—C1—C7—O1	−64.54 (14)	C10'—C11'—C12'—C13'	2.0 (11)
C2—C1—C7—C8	−68.9 (4)	C11'—C12'—C13'—C8'	−2 (2)
C6—C1—C7—C8	116.7 (4)	C9'—C8'—C13'—C12'	2 (3)
O1—C7—C8—C9	168.9 (5)	C12'—C11'—O3'—C15'	8.7 (8)
C1—C7—C8—C9	−12.3 (9)	C10'—C11'—O3'—C15'	−170.5 (5)

Symmetry code: (i) −*x*+5/4, −*y*+1/4, *z*.

### Hydrogen-bond geometry (Å, º)

Cg1 and Cg2 are the centroids of the C8–C13 and C1-C6 rings, respectively

**Table:**

*D*—H···*A*	*D*—H	H···*A*	*D*···*A*	*D*—H···*A*
C16—H16*C*···O1^ii^	0.98	2.51	3.4919 (16)	175
C14—H14*C*···*Cg*1^ii^	0.98	2.83	3.716 (2)	151
C15—H15*B*···*Cg*2^ii^	0.99	2.80	3.6831 (19)	149

Symmetry code: (ii) −*x*+1, *y*+1/4, *z*+1/4.
